# The first cultivated representatives of the actinobacterial lineage OPB41 isolated from subsurface environments constitute a novel order *Anaerosomatales*

**DOI:** 10.3389/fmicb.2022.1047580

**Published:** 2022-11-10

**Authors:** Maria A. Khomyakova, Daria G. Zavarzina, Alexander Y. Merkel, Alexandra A. Klyukina, Valeria A. Pikhtereva, Sergey N. Gavrilov, Alexander I. Slobodkin

**Affiliations:** ^1^Winogradsky Institute of Microbiology, FRC Biotechnology, Russian Academy of Sciences, Moscow, Russia; ^2^Faculty of Biology, Lomonosov Moscow State University, Moscow, Russia

**Keywords:** mud volcano, mineral waters, subsurface biosphere, lithotrophy, thermophile, Fe(III) reduction, protocatechuate, *Coriobacteriia*

## Abstract

The continental subsurface harbors microbial populations highly enriched in uncultured taxa. OPB41 is an uncultured order-level phylogenetic lineage within the actinobacterial class *Coriobacteriia*. OPB41 bacteria have a wide geographical distribution, but the physiology and metabolic traits of this cosmopolitan group remain elusive. From two contrasting subsurface environments, a terrestrial mud volcano and a deep subsurface aquifer, located in the central part of Eurasia, within the Caucasus petroleum region, we have isolated two pure cultures of anaerobic actinobacteria belonging to OPB41. The cells of both strains are small non-motile rods forming numerous pili-like appendages. Strain M08DHB^T^ is mesophilic, while strain Es71-Z0120^T^ is a true thermophile having a broad temperature range for growth (25–77°C). Strain M08DHB^T^ anaerobically reduces sulfur compounds and utilizes an aromatic compound 3,4-dihydroxybenzoic acid. Strain Es71-Z0120^T^ is an obligate dissimilatory Fe(III) reducer that is unable to utilize aromatic compounds. Both isolates grow lithotrophically and consume molecular hydrogen or formate using either thiosulfate, elemental sulfur, or Fe(III) as an electron acceptor. Genomes of the strains encode the putative reductive glycine pathway for autotrophic CO_2_ fixation, Ni-Fe hydrogenases, putative thiosulfate/polysulfide reductases, and multiheme *c*-type cytochromes presumably involved in dissimilatory Fe(III) reduction. We propose to assign the isolated strains to the novel taxa of the species–order levels and describe strain M08DHB^T^ as *Anaerosoma tenue* gen. nov., sp. nov., and strain Es71-Z0120^T^ as *Parvivirga hydrogeniphila* gen. nov., sp. nov., being members of *Anaerosomatales* ord. nov. This work expands the knowledge of the diversity, metabolic functions, and ecological role of the phylum *Actinomycetota*.

## Introduction

One of the least explored environments on Earth, the continental subsurface, harbors microbial populations highly enriched in uncultured taxa. Understanding the ecological roles, adaptation strategies and interspecies interactions of microorganisms whose existence has only been traced by metagenomic studies is hampered without obtaining cultivated laboratory cultures. OPB41 is an uncultured phylogenetic lineage within the phylum *Actinomycetota*. Designation ‘OPB41’ refers to the 16S rRNA gene sequence found by Hugenholtz et al. in Obsidian Pool, a Yellowstone National Park hot spring (Hugenholtz et al., 1998). The SILVA rRNA database places OPB41-related sequences in a separate order-level group within the class *Coriobacteriia*, which currently contains about 2000 entries (Quast et al., 2013).^1^ According to Genome Taxonomy Database^2^, OPB41 also represents a separate order (o_OPB41) within the class *Coriobacteriia* (Parks et al., 2022). No members of OPB41 have been isolated in pure culture or even enriched in selective medium so far. Around 50 complete genomes of OPB41 are publicly available; but studies of the metabolic potential of this bacterial group based on cultivation-independent data are scarce (Bird et al., 2019).

Actinobacteria play important roles in biogeochemical cycling of elements (Goodfellow and Williams, 1983; Holmalahti et al., 1994; Hill et al., 2011). Several actinobacterial species, e.g., from *Nocardia* and *Streptomyces* genera, are capable of aerobic chemolithoautotrophic growth (Aggag and Schlegel, 1973; Gadkari et al., 1990). Anaerobic hydrogenotrophy is a rarer feature of actinobacteria and had only been reported for *Denitrobacterium detoxificans*, a member of *Coriobacteriaceae* family (Anderson et al., 2000). Recently, the genes for key enzymes of the Wood-Ljungdahl pathway for CO_2_ fixation have been identified in MAGs and SAGs of several actinobacteria, including those of *Coriobacteriaceae* family and OPB41 group (Vavourakis et al., 2018; Liu et al., 2020; Merino et al., 2020; Jiao et al., 2021). All of the analyzed genomic data were retrieved from metagenomes of extreme environments, such as serpentinite-hosted systems or hot springs, no putative mesophilic lithotrophs have been proposed so far among OPB41 representatives.

Environmental distribution of ribosomal gene sequences shows that OPB41 is a cosmopolitan group of prokaryotes. We have noticed the presence of OPB41 in the samples from two contrasting types of environments related to deep subsurface – terrestrial mud volcanoes (TMVs) and mineral water aquifers. Our studied sites were located in the central part of Eurasia, within the Caucasus petroleum region, which is subdivided into several petroleum provinces based on their geographic location, shared tectonostratigraphic architecture and petroleum systems elements (Tari et al., 2021).

Terrestrial mud volcanoes are the structures located on the Earth’s surface but geologically connected to deep petroleum fields, and thus, to the subsurface biosphere. Mud volcanism is one of the most exciting geological phenomena with significant implications for hydrocarbon exploration, seismicity, and the atmospheric budget of methane (Mazzini and Etiope, 2017). TMVs can provide direct way to recover subsurface microbial communities due to emission of mud, breccias, liquids, and gases from deep reservoirs to the day surface through fracture networks extending to a depth of several kilometers. TMV fluids generate local physicochemical gradients that allow the proliferation of microorganisms with various metabolic patterns (Khomyakova et al., 2020; Slobodkina et al., 2020; Frolova et al., 2021). Representatives of the phylum *Actinomycetota* are not a major component of TMV’s microbial communities, where methane-oxidizing and sulfur-metabolizing prokaryotes usually predominate (Wang et al., 2014; Lin et al., 2018; Mardanov et al., 2020; Merkel et al., 2021; Tu et al., 2022). However, in several TMVs the relative abundance of *Actinomycetota* reaches 12–15% (Yang et al., 2012; Khomyakova et al., 2022).

Yessentukskoye mineral water deposit (YMWD) located on the territory of the Stavropol Upland petroleum province, is famous for balneologically valuable waters extracted from a kilometer deep Upper Cretaceous (K_2_*s*-*m*) aquifer. The YMWD is characterized by the proximity of the crystalline basement and heating zones to its aquifers, the presence of igneous intrusions (laccoliths) embedded in the whole thickness of water-bearing sedimentary rocks, and tectonic faults serving as well-permeable conduits between the basement and the sedimentary cover. This geological complexity leads to the formation of highly diverse mineral waters with salinity ranging from 0.5 to 14.0 g l^−1^, temperatures ranging from 10 to ~70°С, and variation of predominant ions (carbonate, bicarbonate, chloride or sulfate; Abramov and Vavichkin, 2010; Filimonova et al., 2020; Lavrushin et al., 2020). We have previously detected the representatives of the phylum *Actinomycetota* in waters extracted from two production wells of YMWD. The wells penetrated K_2_*s*-*m* aquifer where the abundance of uncultured actinobacterial groups reached 30% (Gavrilov et al., 2022).

Here we report the physiological and genomic characterization of two pure cultures of anaerobic bacteria belonging to OPB41 group, isolated from subsurface environments of the Caucasus petroleum region, where they constitute significant parts of two different microbial communities. The isolates are physiologically dissimilar from each other, yet share a common metabolic feature of anaerobic hydrogenotrophic or formatotrophic lithotrophy.

## Materials and methods

### Sampling site and cultivation conditions

Strain M08DHB^T^ was isolated from a mixed sample of sediment and water named B03 and collected in September 2018 from a salsa lake of terrestrial mud volcano Karabetova Gora (45.202 N, 36.783 E), Taman Peninsula, Krasnodar kray, Russia. Temperature and pH at the sampling site were 15°C and 8.0–8.5, respectively, salinity was 6.0 g l^−1^. The water contained 24.3 mM chloride and 1.2 mM sulfate. Dissolved sulfide, nitrate, and nitrite were under the detection level (<0.01 mM). The water did not contain soluble iron ions, but the sediment had high HCl-extractable iron content (81.9 mM Fe(II) and 2.7 mM Fe(III)), due to the presence of iron-bearing minerals.

Enrichment and isolation were performed in the liquid medium of the following composition (per liter distilled water): KH_2_PO_4_, NH_4_Cl, KCl, MgCl_2_·6H_2_O (0.33 g each), CaCl_2_·6H_2_O, 0.033 g, NaHCO_3_, 2.0 g, Na_2_S·9H_2_O, 0.5 g, 1 ml trace element solution (Slobodkin et al., 2012), 1 ml vitamin solution (Wolin et al., 1963) and 1 ml resazurin solution (up to 0.001 g l^−1^ in the medium). The medium was prepared by boiling and cooling it under N_2_:CO_2_ (80:20) flow, afterwards, NaHCO_3_, vitamins, and Na_2_S·9H_2_O were added. Sodium sulfide was used as a reducing agent. Resazurin was added as a redox indicator. The medium was dispensed in 10 ml aliquots into 17 ml Hungate tubes; the headspace was filled with N_2_:CO_2_ (80:20, high-purity grade). The medium was autoclaved at 1 atm, 121°C for 20 min. The pH of the sterile medium was adjusted to 7.0–7.5 at 25°C with 10% sterile anaerobic NaOH solution using anaerobic technique. 3,4-dihydroxybenzoic (protocatechuic) acid and MgSO_4_.7H_2_O from sterile anoxic stock solutions were added before inoculation to a final concentration of 10 mM each.

Strain Es71-Z0120^T^ was isolated from water sampled in September 2020 from the well 71 of YMWD that is utilized for industrial extraction of Yessentuki no. 4 type mineral water. This well (44.189 N, 42.942 E) has a maximal depth of 999 m with open boreholes in the interval of 676.0–998.9 m. Temperature and pH values of water at the wellhead were 40.5°C and 6.8–6.9, respectively, salinity was 7.7 g l^−1^. The water contained 71.8 mM bicarbonate and 47.4 mM chloride, as well as 10.4 ppb dissolved iron ions. Dissolved sulfate, sulfide, nitrate, and nitrite were under the detection level (<0.01 mM). Water-bearing sediments of the aquifer penetrated by the well 71 contained the iron minerals siderite and glauconite, according to the local geological survey data.

Sterile 17 ml Hungate anaerobic culture tubes, pre-filled with 100% CO_2_ gas, synthesized ferrihydrite (SF), and acetate were used to obtain enrichment cultures. 10 ml of fresh water, sampled from the wellhead, were injected into the tubes with sterile syringes and needles, and the tubes were further incubated in the dark at 47°C, the average temperature observed during well operation in tap mode. Thus, water samples served as both the basic mineral medium and the inoculum for primary enrichments. The initial content of Fe(III) and acetate in the primary enrichments comprised 10 mM each. The basic cation-anion composition of the medium used for pure culture isolation corresponded to that previously determined for Yessentuki no. 4 type mineral water (Zavarzina et al., 2022). The medium contained (per liter distilled water): KH_2_PO_4_, NH_4_Cl, KCl, MgCl_2_·6H_2_O (0.33 g each), CaCl_2_·6H_2_O, 0.033 g, NaCl, 2.00 g, NaHCO_3_, 6.00 g, Na_2_S·9H_2_O, 0.01 g, 1 ml trace mineral solution (Kevbrin and Zavarzin, 1992), 1 ml vitamin solution (Wolin et al., 1963). Sodium formate 1 g l^−1^, or H_2_ (10% v/v) was used as the electron donor and SF as the electron acceptor. SF was prepared as described previously (Zavarzina et al., 2006) and added to the culture medium up to the final Fe(III) content of 10 mM, before sterilization. The medium was prepared by boiling and cooling it under CO_2_ flow, afterwards, NaHCO_3_, vitamins, and Na_2_S·9H_2_O were added. The medium was dispensed in 10 ml aliquots into 17 ml Hungate tubes, the headspace was filled with extra pure CO_2_. The medium was autoclaved at 1 atm, 121°C for 20 min. The pH of the medium after sterilization was 6.8 at room temperature.

### Phenotypic characterization of the strains

Growth of the isolates was monitored by direct cell counting using a phase-contrast microscope (Olympus CX-43) for the cultures of strain M08DHB^T^, and a fluorescence microscope Axio Lab.A1 (Zeiss, Germany) for the cultures of strain Es71-Z0120^T^ whose subsamples were pre-stained with acridine orange dye for DNA. Transmission electron microscopy was performed to determine cell morphology using JEM-100 and JEM-1400 electron microscopes (JEOL, Japan) at the UNIQEM Collection Core Facility, FRC Biotechnology of the Russian Academy of Science. Morphology of the cultures with iron minerals was examined by scanning electron microscopy (SEM) using TESCAN VEGA 3 LMU device with an INCA Energy 350/X-max 80 energy-dispersive analysis system (OXFORD Instruments NanoAnalysis, United Kingdom). Specimens were pre-fixed with a carbon double-sized scotch tape and triply coated with Au.

All the cultivation experiments were performed in duplicate using Hungate tubes, 1 ml inoculum volume was used for the transfers of the strain M08DHB^T^, and 0.5 ml inoculum volume was used for the strain Es71-Z0120^T^. Temperature (from 10 to 60°C) and pH growth ranges of the strain M08DHB^T^ were determined using the same medium as for the strain isolation, with formate and thiosulfate serving as the electron donor and acceptor, respectively. The pH of the medium was adjusted to different values in the range of 5.0–10.0 with sterile anaerobic solutions of HCl (2 M) or NaOH (1.25 M). The NaCl requirement for growth was determined in a medium of similar mineral composition, but lacking NaCl and containing 0.33 g l^−1^ MgCl_2_·6H_2_O. Varying amounts of NaCl (0–100 g l^−1^) were added directly into Hungate tubes from pre-sterilized anaerobic stock solutions.

To determine optimal growth conditions of strain Es71-Z0120^T^, temperatures ranging from 10 to 78°C, NaCl concentrations up to 25g l^−1^, sodium bicarbonate concentrations up to 30 g l^−1^, and pH ranging from 5.5 to 8.15 were tested with formate and Fe(III)-citrate (10 mM) as the electron donor and acceptor, respectively. In these cases, sodium sulfide was omitted from the medium. Cell counts were performed within 5–20 days of incubation. Optimal NaCl concentrations for growth were determined on a modified medium in which all chlorides, other than NaCl, were replaced with equimolar concentrations of sulfates. pH optimum of strain Es71-Z0120^T^ was determined on a medium containing 3.0 g l^−1^ NaHCO_3_, prepared under extra pure CO_2_ gas flow. 6 M HCl solution was used for pH adjustment to the values below 6.1, while different pH values in the range of 6.1–7.0 were reached by the gradual replacement of CO_2_ with N_2_ in the gas phase. Solution of Na_2_CO_3_ (100 g l^−1^) and pure N_2_ in the gas phase were used to sustain pH values above 7.0.

For both strains, aerobic and microaerobic growth was tested using the media lacking the reducing agent, under 2, 4, or 10% O_2_ (v/v in CO_2_) or 100% air in the gas phase. Catalase activity was tested by the bubble production assay with 3% (v/v) solution of H_2_O_2_. Oxidase activity was determined with 10 g l^−1^ tetramethyl-*p*-phenylenediamine (Cappuccino and Sherman, 2002). Electron donors and acceptors were added from sterile anaerobic stock solutions before inoculation. All the organic substrates (peptides, carbohydrates, alcohols, and organic acids) were filter-sterilized using 0.2 μm pore size syringe filters (Millipore) and added to a final concentration of 3 g l^−1^ or 0.3% (v/v). Growth with molecular hydrogen (100% or 10% in the gas phase) as the electron donor and utilization of non-fermentable substrates were tested in the presence of an optimal electron acceptor. Formate concentration was monitored on a Stayer HPLC (Aquilon) equipped with a refractometric detector (Knauer) and an Aminex HPX-87H column (Bio-Rad), operated isocratically, with 5 mM H_2_SO_4_ as an eluent at 0.6 ml min^−1^. Subsamples of the cultures for chromatographic analysis were prepared by centrifugation at 12100 g for 3 min and further pH adjustment of the supernatants to 2.0 with 5 mM H_2_SO_4_. Molecular hydrogen consumption and the formation of gaseous metabolites were monitored by gas chromatography (GC) on a HayeSep N 80/100 mesh column at 40°C with argon as the carrier gas at 20 ml min^−1^ flow rate. Sulfide concentration was determined colorimetrically with dimethyl-*p*-phenylenediamine (Trüper and Schlegel, 1964). Fe(II) production during the growth with Fe(III) compounds was monitored with ferrozine (Stookey, 1970), Fe(II) from SF and magnetite was preliminary extracted with 0.6 M HCl. 9,10-anthraquinone-2,6-disulfonate (AQDS) reduction was monitored by the change in the medium color.

Chemotaxonomic analysis was performed with the cells grown on formate with thiosulfate (for strain M08DHB^T^) or ferric citrate (for strain Es71-Z0120^T^) serving as the electron acceptors. For fatty acid analysis, the isolates were cultivated at optimal physicochemical growth conditions. Biomass was collected in the late exponential growth phase. Cellular fatty acid profiles were determined by GC–MS using methyl ester derivatives prepared from 5 mg of freeze-dried biomass treated with anhydrous HCl/MeOH. The determination was based on the retention time, reference equivalent chain length values (Hartig, 2008), and mass spectra using Supelco standards and NIST MS Search 2.0 program provided with the GC–MS setup. Cellular fatty acids content was determined as percentages of the total ion current peak area. Quinones were analyzed as described by Collins et al. (Collins and Jones, 1981; Collins, 1985) using Finnigan 123 LCQ Advantage MAX APSI/MS ion trap mass spectrometer.

### 16S rRNA gene sequencing and analysis

Genomic DNA isolation and 16S rRNA gene amplification and sequencing were performed as described previously (Slobodkina et al., 2016). The GenBank accession numbers for 16S rRNA gene sequences of strains M08DHB^T^ and Es71-Z0120^T^ are ON668121 and OP389241, respectively. The 16S rRNA gene sequences of the isolates were compared with other sequences in GenBank (Benson et al., 1999) by using the BLAST program (Altschul et al., 1990) and by means of the EzBioCloud server^3^ (Yoon et al., 2017) to identify their closest relatives. Sequences were aligned by MAFFT v7.427 (G-INS-i strategy; Nakamura et al., 2018) for 16S rRNA gene-based phylogenetic analyses.

### Genome sequencing and analysis

Complete genome sequencing of both strains was carried out on a MiSeq system (Illumina, San Diego, California, United States) using the reagent kit providing for 2 × 250 bp readings. Whole Genome Shotgun project has been deposited at DDBJ/ENA/GenBank under the accession JALNTY010000000 for M08DHB^T^ and JAMCCO010000000 for Es71-Z0120^T^. Gene search and annotation were performed by BLAST (Altschul et al., 1990) and IMG (Chen et al., 2021) services. AAI values were calculated using an online tool developed by the Kostas group at the Georgia Institute of Technology (Rodriguez-R and Konstantinidis, 2016). For genome-based phylogenetic reconstructions, 120 bacterial single-copy conservative marker genes were used as described previously (Parks et al., 2020). The trees were built using the IQ-TREE 2 program (Minh et al., 2020) with fast model selection *via* ModelFinder (Kalyaanamoorthy et al., 2017) and ultrafast approximation for phylogenetic bootstrap (Hoang et al., 2018), as well as approximate likelihood-ratio test for branches (Anisimova and Gascuel, 2006).

## Results

### Enrichment and isolation

#### Strain M08DHB^T^

Analysis of the prokaryotic diversity in salsa lake sediments of Karabetova Gora mud volcano by high-throughput sequencing of 16S rRNA gene amplicons showed that representatives of OPB41 group constituted 13–14% of all reads (Khomyakova et al., 2022). An enrichment culture was initiated by 10% (w/v) inoculation of a mud sample into anaerobic sterile medium with 3,4-dihydroxybenzoic (protocatechuic) acid and sulfate. Protocatechuic acid was chosen as a biodegradable substrate, modelling the aromatic hydrocarbons present in mud volcanoes (Remizovschi et al., 2020). After 3 weeks of incubation at 30°C in the dark the growth of small thin rods was observed. After three subsequent 10% (v/v) transfers, the enrichment was partially purified (up to 60%) by serial 10-fold dilutions in the same liquid medium (Supplementary Figure S1). Well-separated brownish round colonies (1–2 mm in diameter) appeared on Gelrite blocks after 14–17 days of further incubation on 3,4-dihydroxybenzoic acid. When cultivated with formate and thiosulfate, the isolate formed whitish microcolonies, invisible to the naked eye. Individual colony from the 10^−6^ dilution was transferred to a test tube with liquid medium, after 4–7 days of incubation, microbial growth was observed, and this culture, designated strain M08DHB^T^ was used for further studies.

#### Strain Es71-Z0120^T^

16S rRNA gene sequence profiling of different microbial communities of YMWD mineral waters retrieved relative abundance of the representatives of OPB41 group in the range of 3–9% of all prokaryotic reads (Supplementary Figure S1). An enrichment culture from well 71 was initiated by incubation of a freshly taken water sample into a test-tube supplemented with acetate and ferrihydrite under 100% CO_2_ in the gas phase. Active microbial growth was observed after 4 days of incubation being accompanied by the transformation of brown-colored precipitate of SF into a black magnetic mineral, presumably magnetite. Microscopic observations of acridine orange-stained samples of the primary enrichment revealed the predominance of vibrio-like cells in the culture and lower representation of very small rods. Both morphotypes were closely associated with iron mineral particles. 16S rRNA gene fragment profiling of the primary enrichment revealed the predominance of novel representatives of the order *Deferribacterales* (79% relative abundance), and a small share of OPB41 group uncultured actinobacteria (3%). Acetate replacement with formate led to the substitution of the predominating morphotype in the enrichment with small rods. 16S rRNA gene fragment profiling of the novel culture indicated increased representation of an OPB41-related phylotype (58%). This bacterium was isolated into a pure culture by serial ten-fold dilutions. The culture in the last positive dilution (10^−7^) contained morphologically homogeneous non-motile small rod-shaped cells and was designated strain Es71-Z0120^T^. The purity of the culture was assessed by routine microscopic examination and confirmed by complete 16S rRNA gene and complete genome sequencing.

### Phylogenomic analysis

**Figure 1:**
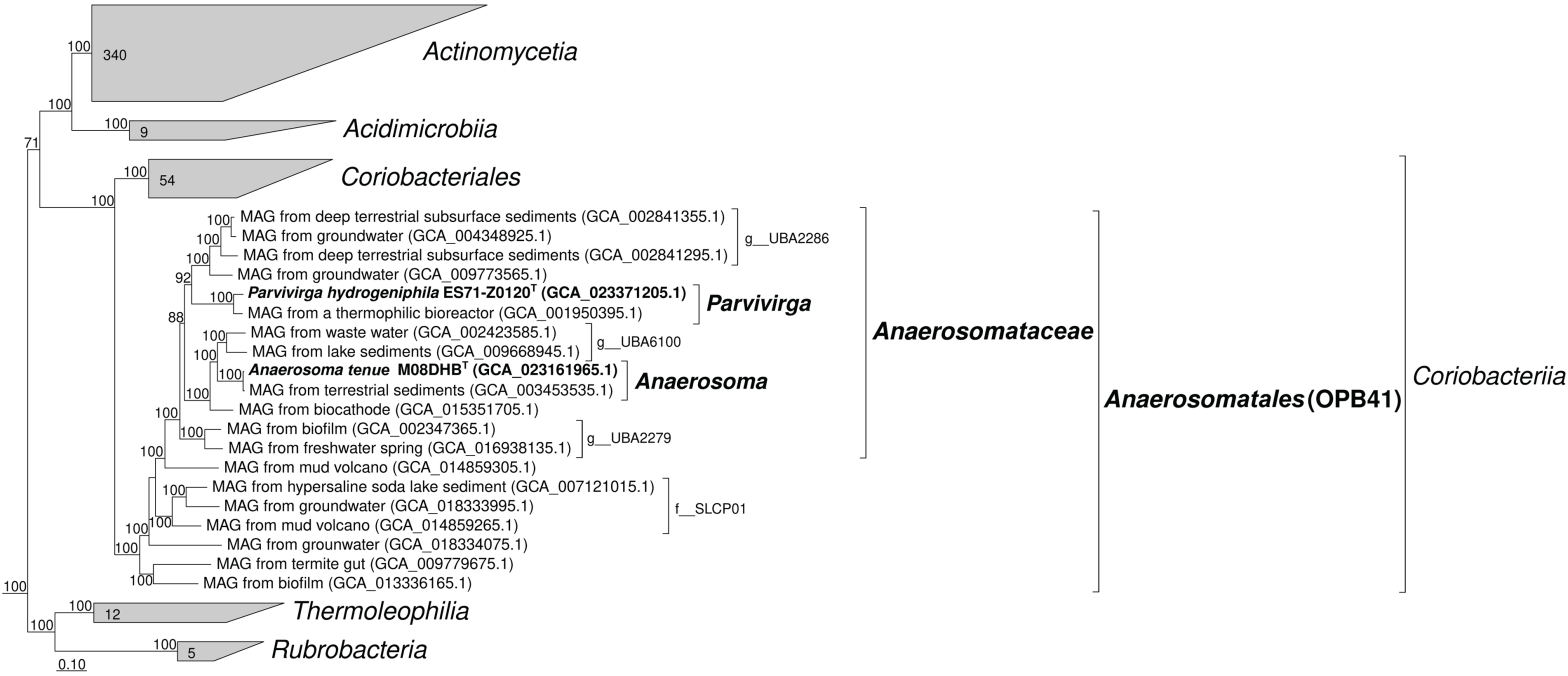
Phylogenomic placement of strains M08DHB^T^ and Es71-Z0120^T^ within the *Actinomycetota* phylum based on concatenated partial amino acid sequences of 120 bacterial single copy conserved marker genes with taxonomic designations according to the Genome Taxonomy DataBase. Bootstrap consensus tree is shown with values above 70%, placed at the nodes. Bar, 0.1 changes per position.

According to the results of our phylogenomic analysis based on concatenated partial amino acid sequences of 120 bacterial single-copy conserved marker genes (Figure 1), both new strains belong to the phylogenetic lineage OPB41 of the order rank, and represent separate genera in a single family-level lineage, which is designated as “UBA2279” in the Genome Taxonomy DataBase, and for which we propose the name ‘*Anaerosomataceae*’ (see below). Division of M08DHB^T^ and Es71-Z0120^T^ isolates into two genera is strongly supported by AAI comparison, which retrieved 54.4–57.7% identity of one-way AAI and 62.5% identity of two-way AAI, as well as by 94.3% 16S rRNA gene sequence identity. On a 16S rRNA-based tree, M08DHB^T^ and Es71-Z0120^T^ isolates also form a separate group within class *Coriobacteriia*, although statistical support for this separation is not strong (Supplementary Figure S2).

### Environmental distribution of OPB41-related phylotypes

The survey of the environmental distribution of publicly available 16S rRNA gene sequences, related to the family-level lineage ‘*Anaerosomataceae’* (Figure 1), shows that similar shares of sequences were retrieved from soils and different oil-bearing environments, such as petroleum crude oil, oil sands, oil wells, oil-contaminated wastewaters and tailings. A lesser share of this group sequences was retrieved from various sedimentary and bottom water environments, including the sediments of pristine waterbodies, soda lakes and marine shallow waters (Supplementary Figure S3 A). The majority of MAGs related to ‘*Anaerosomataceae*’ were retrieved from oil-bearing ecosystems (Supplementary Figure S3 B). Somewhat dissimilar result was obtained when all the OPB41-related MAGs were considered for the analysis. The number of MAGs retrieved from bottom waters and sediments was almost twice as much as the number of those assembled from the samples of oil-containing environments or subsurface habitats, not associated with oil and gas deposits (19 v/s 10 and 12 sequences, respectively, Supplementary Figure S4). Totally, the majority of publicly available OPB41-related phylotypes were detected in oil-bearing, sedimentary and subsurface environments.

### Phenotypic characterization of novel isolates

**Figure 2:**
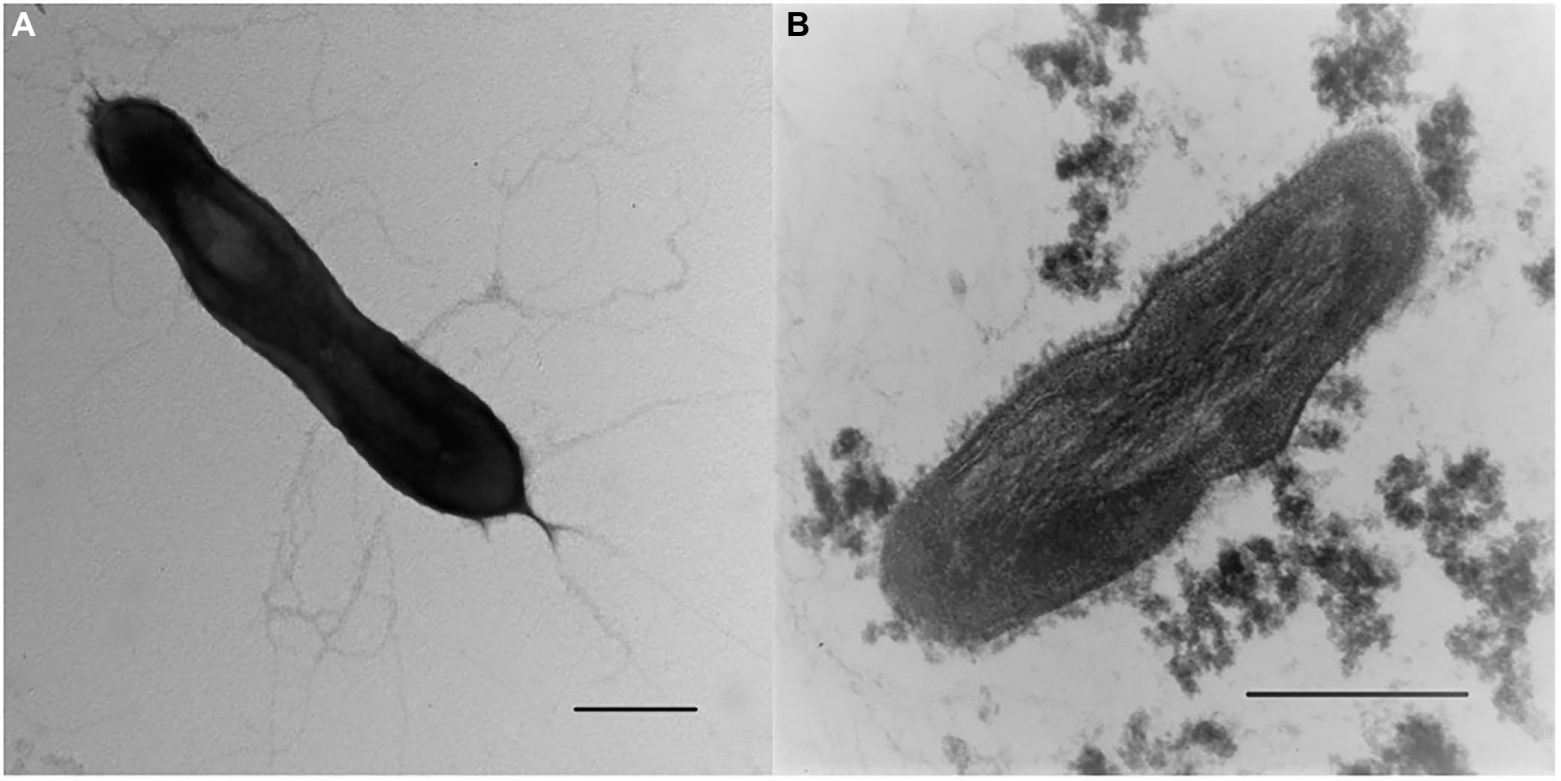
Cellular morphology of strain M08DHB^T^. **(A)** Electron micrograph of negatively stained cells showing an overall cell morphology and localization of filamentous appendages around a cell. Bar, 0.2 μm. **(B)** Ultrathin section of a cell, showing thick bilayered cell-wall structure. Bar, 0.2 μm.

**Figure 3:**
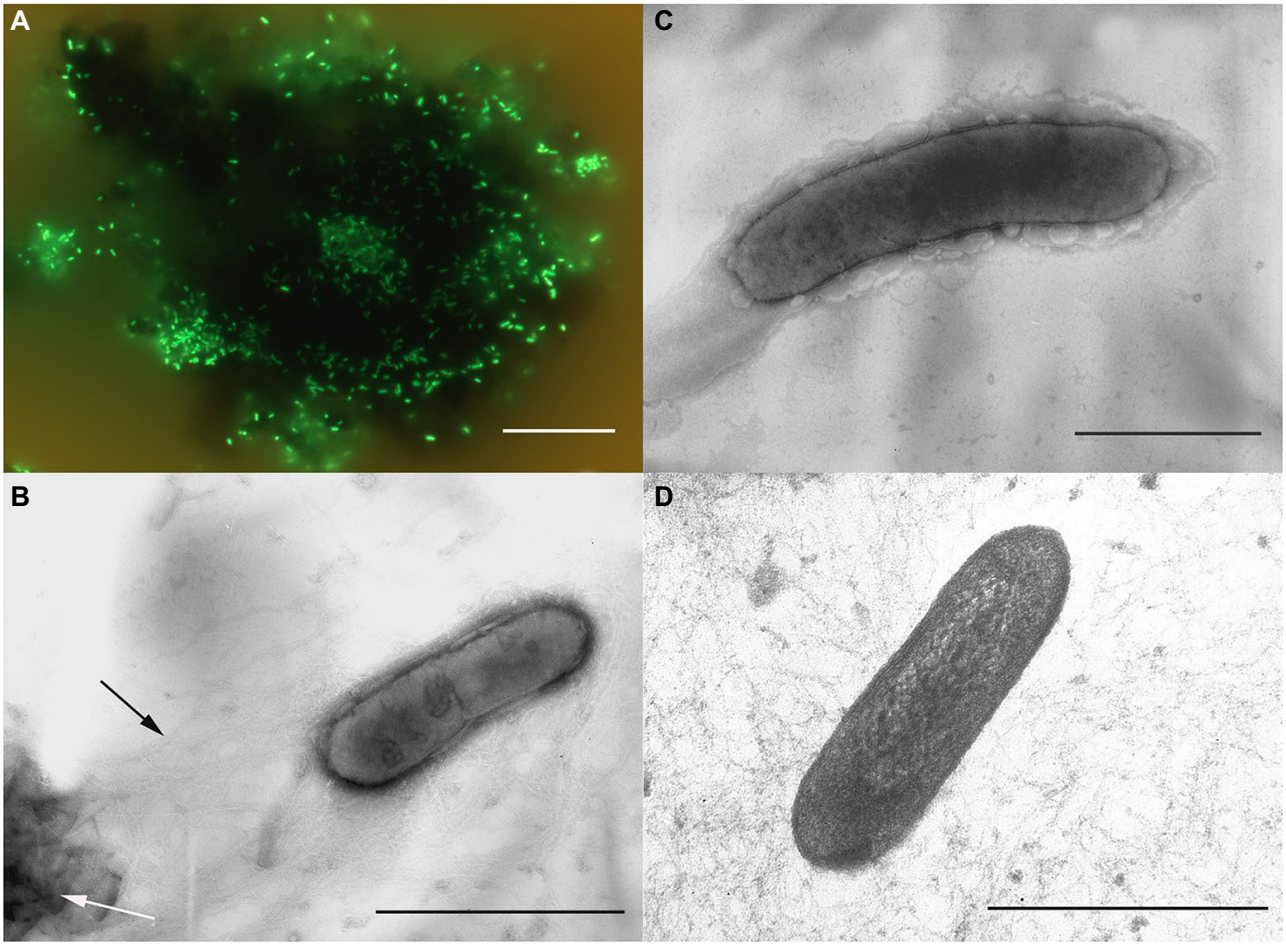
Cellular morphology of strain Es71-Z0120^T^. **(A)** Acridine orange-stained cells colonizing an SF particle. **(B)** Electron micrograph of a negatively stained cell grown with SF. *Black arrow* indicates a network of pili-like appendages, *white arrow* points at a mineral particle of SF. **(C)** Electron micrograph of a negatively stained cell grown with soluble Fe(III)-citrate. **(D)** Ultrathin section of a cell. Bars: a = 10 μm, b–d = 0.3 μm.

Morphological, physiological and chemotaxonomic characteristics of strains M08DHB^T^ and Es71-Z0120^T^ are summarized in Table 1. The cells of the isolates are small, non-motile rods with Gram-positive cell wall type (Figures 2, 3). Both strains have pili-like appendages that cover the entire cell surface. The pili of strain Es71-Z0120^T^ are 0.8–0.9 nm in diameter and appear only in the presence of ferrihydrite, but not in the presence of soluble Fe(III)-citrate (Figures 3B,C). The cells of strain Es71-Z0120^T^ usually form dense clusters strongly associated with ferrihydrite particles (Figure 3A; Supplementary Figure S5 A). In the late stationary growth phase or during long storage, cells of strain Es71-Z0120^T^ form dense extracellular matrix. This matrix stains green with acridine orange dye and is visualized under a fluorescent microscope as large bubble-like structures interconnected by long strands from which small bubbles of various diameters bud off (Supplementary Figures S5 B,D). Energy dispersive X-ray spectroscopy (EDS) revealed that the ‘bubbles’ observed in the late stationary growth phase consist mainly of oxygen and carbon but are enriched with silicon and iron comparing to the cells themselves (Supplementary Figures S5 D, S6B). Scanning electron microscopy revealed that siderite is likely to be the main mineral product of ferrihydrite reduction (Supplementary Figures S5 C, S6A) by the culture of Es71-Z0120^T^.

**Table 1:** Morphological, physiological and chemotaxonomic characteristics of strains M08DHB^T^ and Es71-Z0120^T^.

Characteristic	Strain M08DHB^T^	Strain Es71-Z0120^T^
Cell morphology and motility	Straight to slightly curved non-motile singular rods (Figure 2A)	Straight or slightly curved non-motile singular rods (Figures 3B–D)
Cell size, length x diameter, μm	0.8–1.4 × 0.14–0.18	0.5–1.5 × 0.15–0.2
Cell wall type	Gram-positive (Figure 2B)	Gram-positive (Figure 3C)
Endospore formation	No	No
Aerobic (20% O_2_) or microaerobic (2% O_2_) growth	No	No
Temperature range, min – optimum – max	14–30 – 42	25–47-60 – 77
pH range, min – optimum – max	6.0–7.0-7.5 – 8.5	6.0–6.8-7.2 – 8.5
NaCl concentration range, g l^−1^, min – optimum – max	0–5.0-10 – 70	0–0-0.5 – 35
NaHCO_3_ concentration range, g l^−1^, min – optimum – max	Not applicable	0–2.0 – 10
Electron donors utilized	3,4-dihydroxybenzoic acid, formate, H_2_	formate, H_2_
Electron acceptors utilized	Elemental sulfur, thiosulfate	Ferrihydrite, Fe(III)-citrate
Yeast extract requirements	At least 0.05 g l^−1^ was required for growth on any of the utilized substrates	Not required
Doubling time under optimal growth conditions	4 h (formate with thiosulfate)	3 h (formate with Fe(III)-citrate)
Cellular fatty acids*	С18:0 (27%), C16:0 (24%), С18:1 n-9 (22%)	C18:2 n-6 (45%), C18:1 n-9 (22%), C16:0 (13%)
Respiratory quinones	Not detected	Not detected

* *Three major fatty acids are indicated. Refer to* *Supplementary Table S1* *for the complete list*.

**Figure 4:**
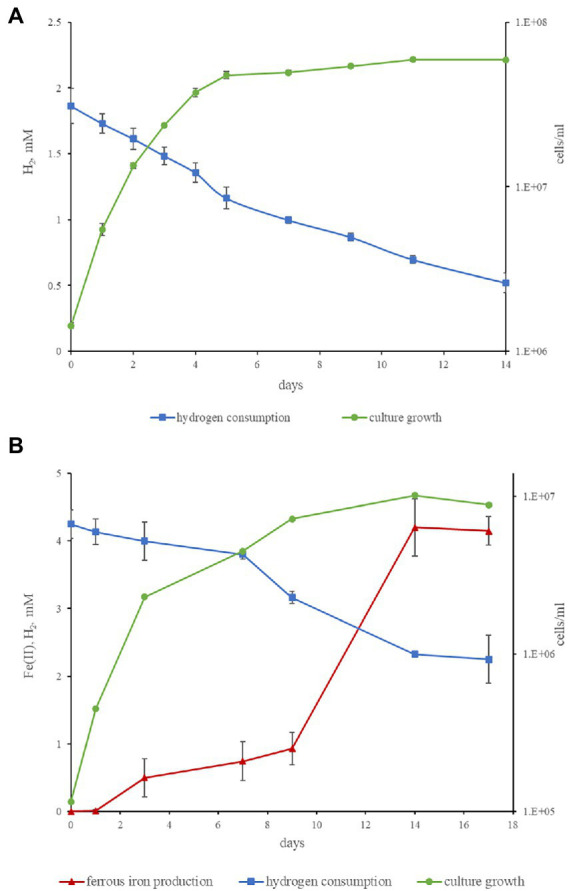
Kinetics of hydrogenotrophic growth of strains M08DHB^T^ and Es71-Z0120^T^. **(A)** Growth curve of strain M08DHB^T^ on the medium with hydrogen and thiosulfate in the presence of yeast extract, hydrogen consumption by the strain. **(B)** Growth curve of strain Es71-Z0120^T^ on the medium with hydrogen and SF, hydrogen consumption and Fe(III) reduction. Note different cell yields of the strain M08DHB^T^ growing lithoheterotrophically with yeast extract and the strain Es71-Z0120^T^ growing lithoautotrophically with insoluble electron acceptor ferrihydrite.

Both strains are obligate anaerobes, no growth was observed at 2% or 20% O_2_ in the gas phase, but the strains were positive for catalase and oxidase tests. Strain M08DHB^T^ is mesophilic, while strain Es71-Z0120^T^ is thermophilic and can grow in a wide temperature range from 25 to 77°C, with broad temperature optimum of 47–60°C. Strains M08DHB^T^ and Es71-Z0120^T^ use a limited number of electron donors and acceptors for their growth. The common features of both isolates are hydrogenotrophy and formatotrophy. The strains are capable of lithotropic growth with molecular hydrogen and an inorganic electron acceptor (Figure 4). Strain M08DHB^T^ grows with molecular hydrogen in the presence of thiosulfate and requires 50 mg l^−1^ of yeast extract and vitamins for its lithotrophic growth (Figure 4A). Strain Es71-Z0120^T^ grows lithoautotrophically with ferrihydrite in the presence of vitamins and in the absence of yeast extract (Figure 4B). Under these growth conditions, the highest cell density of strain Es71-Z0120^T^ was observed at low hydrogen content (5% v/v in the gas phase) with 10 mM Fe(III) supplied as ferrihydrite mineral (SF). The growth was accompanied by stoichiometric H_2_ consumption and Fe(II) production in the form of siderite (Figure 4B). Both strains consume *ca.* 1.5 mM H_2_ within 14 days of lithotrophic growth at optimal conditions. In the presence of thiosulfate or S^0^ strain М08DHB^T^ oxidizes formate to CO_2_ and produces sulfide. Acetate production was not detected. In contrast, the growth of strain Es71-Z0120T^T^ on 10 mM formate under ferrihydrite-reducing conditions is accompanied by the production of acetate in trace amounts. Both strains do not grow on formate or hydrogen without an electron acceptor, as well as on vitamins or yeast extract in the absence of hydrogen or formate. However, strain M08DHB^T^ utilizes 3,4-dihydroxybenzoic acid in the absence of electron acceptors but in the presence of 50 g l^−1^ yeast extract. Metabolic products of growth on this substrate are acetate, CO_2_ and traces of H_2_. Strain Es71-Z0120T^T^ does not grow on 3,4-dihydroxybenzoic acid in the presence or absence of yeast extract or electron acceptors.

### Genomes statistics

The draft genome of strain M08DHB^T^ was assembled into 6 contigs with genome size of 2,107,022 bp and the DNA G + C content of 66.58%. The genome was predicted to contain 2,100 protein-coding sequences and 53 RNA genes including 3 rRNA and 46 tRNA. NCBI PGAP genome annotation tools identified 2038 genes and 9 pseudogenes. The draft genome of strain Es71-Z0120^T^ was assembled into 9 contigs with genome size of 1,840,996 bp and the DNA G + C content of 67.65%. The genome contained 1,808 protein-coding sequences and 54 RNAs genes, including a single copy of 16S rRNA gene. Detailed genomes statistics can be found in Supplementary Table S2. We have analyzed the genomic data in relation to central carbon and energy metabolism of the novel isolates.

### Central metabolism

Genes of the proton-translocating type I NADH-dehydrogenase are present in both strains but in differentially organized loci. Strain M08DHB^T^ possesses the genes encoding 10 subunits of the respiratory complex I in a locus MCK8114634-44 in the order *nuoNMLKJIHDCBA*. Strain Es71-Z0120^T^ possesses two loci, MCL4079026-34 encoding 8 subunits NuoACDHJKLM of the complex I, and MCL4079286-96 encoding 10 subunits NuoNMLKJIHDCBA. Both strains lack the genes encoding the NuoEFG subunits known to form the domain involved in NADH binding and oxidation (Sazanov, 2007). Thus, the respiratory complexes I of these organisms are likely to accept electrons from alternative donors besides NADH (e.g., reduced ferredoxin). Genes for the respiratory complex II, and canonical or alternative complexes III are absent from the genomes of both strains. Oxidative phosphorylation in these organisms is determined by canonical F_0_F_1_-type ATP-synthases encoded in the loci MCK8114601-8 and MCL4078772-9 in the strains M08DHB^T^ and Es71-Z0120^T^, respectively. Alternatively, both organisms could synthetize ATP *via* bacterial A/V-type ATPases which are supposed to be bifunctional (Stewart et al., 2014). These enzyme complexes are encoded in the loci MCK8114895-903 (*atpDBAFCEKI* genes) and MCK8115595-602 (*atpDBAFCEL*) in the strain M08DHB^T^. Similar order of genes encoding A/V-type ATPases is observed in the loci MCL4079475-83 and MCL4078579-85 of Es71-Z0120^T^ strain genome.

The inability of strains M08DHB^T^ and Es71-Z0120^T^ to utilize sugars, which are typical energy and carbon sources for actinobacteria, is obviously caused by the absence of glucose or other sugar transporter genes in both genomes. In addition, both strains lack the genes of phosphofructokinase or glucokinase but possess fructose-1,6-bisphosphatase genes instead, which provide for the irreversible transformation of fructose-1,6-bisphosphate to fructose-6-phosphate in gluconeogenesis direction of glucose metabolism. All the other genes determining the glycolysis/gluconeogenesis pathway are present in the genomes of strains M08DHB^T^ and Es71-Z0120^T^, starting from pyruvate:ferredoxin oxidoreductases encoded in the loci MCK8115834-35 and MCL4079529-30, respectively, and ending with phosphoglucomutases encoded by MCK8114681 and MCL4079339. Both these proteins share considerable homology (32% amino acid sequence identity at an e-value 10^−36^ and complete query sequence coverage) with structurally characterized archaeal phosphoglucomutase of *Thermococcus kodakaraensis*. Interestingly, the interconversion of 2-and 3-phosphoglycerate is catalyzed in the strains M08DHB^T^ and Es71-Z0120^T^ by 2,3-bisphosphoglycerate-independent phosphoglycerate mutase (iPGAM) which is a metalloenzyme distantly related to archaeal iPGAM and distinct from the unrelated cofactor-dependent bacterial dPGAM (Jedrzejas et al., 2000). This enzyme is encoded by MCK8113924 in strain M08DHB^T^ and by MCL4078879 in strain Es71-Z0120^T^. In both our isolates, the products of gluconeogenesis could enter the metabolic network as glucose-1-phosphate *via* uridylyltransferase reaction and further transformation of UDP-glucose, or as glucose-6-phosphate *via* the reactions of incomplete pentose phosphate pathway. Both isolates possess incomplete gene sets determining the TCA cycle. The genomes encode citrate synthetases, aconitate hydratases, isocitrate dehydrogenases, 2-oxoglutarate dehydrogenases, succinyl-CoA synthetases, fumarate hydratases. Genes encoding succinate dehydrogenases/fumarate reductases are absent from both genomes. Strain M08DHB^T^ also lacks malate dehydrogenase genes but malate could be formed from pyruvate by the NADP-dependent malic enzyme encoded by MCK8114652.

In both strains, acetyl-CoA, formed within autotrophic CO_2_ fixation, can enter the gluconeogenesis pathway *via* pyruvate:ferredoxin oxidoreductase, and the TCA cycle *via* the citrate synthetase. Oxaloacetate can be produced from pyruvate by the pyruvate carboxylase encoded in the locus MCK8114401-2 in strain M08DHB^T^, and in the locus MCL4079139-40 in strain Es71-Z0120^T^. Alternatively, acetyl-CoA could be converted to acetate by acetate-CoA ligase encoded in the loci MCK8114182 and MCL4079557.

### Autotrophic CO_2_ fixation

In the genomes of strains M08DHB^T^ and Es71-Z0120^T^, we did not find genes encoding the key enzymes of six microbial carbon fixation pathways, *viz.* ribulose 1,5-bisphosphate carboxylase (Calvin-Benson cycle), carbon monoxide dehydrogenase/acetyl-CoA synthase complex (reductive acetyl-CoA pathway), ATP-citrate lyase and citryl-CoA lyase (two variants of the reductive tricarboxylic acid cycle), 4-hydroxybutyryl-CoA dehydratase (3-hydroxypropionate/4-hydroxybutyrate and dicarboxylate/4-hydroxybutyrate cycles), and malonyl-CoA reductase (3-hydroxypropionate bi-cycle). The absence of succinate dehydrogenase genes from both genomes indicates the inability of our isolates to use the reversed oxidative tricarboxylic acid cycle for CO_2_ assimilation (Mall et al., 2018; Nunoura et al., 2018).

We assume that CO_2_ fixation in strains M08DHB^T^ and Es71-Z0120^T^ proceeds *via* the reductive glycine pathway (Sánchez-Andrea et al., 2020). Genomes of both strains contain a similar set of genes providing autotrophic carbon assimilation through glycine formation. The molybdenum-containing formate dehydrogenase (MCK8114746-7; MCL4079382-3) reduces CO_2_ to formate. Formate-tetrahydrofolate ligase (MCK8114519; MCL4079206) catalyzes the condensation of formate with tetrahydrofolate (THF) and, together with the methylene-THF dehydrogenase/cyclohydrolase (MCK8114455; MCL4079205), converts formate to methylene-THF. Glycine cleavage system (MCK8114290-3; MCL4079664-7), operating in the reductive carboxylation direction, produces glycine from CO_2_, methylene-THF and ammonia. Glycine could be further transformed to acetyl-phosphate with participation of thioredoxin-disulfide reductase (MCK8115865-6; MCL4078363-4) or to serine by serine hydroxymethyltransferase (MCK8114613; MCL4078767). Acetyl-phosphate can be converted to acetyl-CoA by phosphoacetyltransferase (MCK8114184; MCL4079559) or to acetate by acylphosphatase (MCK8115765; MCL4078211). Serine could be assimilated into the central metabolism employing L-serine dehydratase (MCK8115376-7; MCL4078168-9) to form pyruvate. Ammonia, necessary for the formation of glycine, is delivered to the cytoplasm of strain M08DHB^T^ using specific transporter (MCK8115391). Strain Es71-Z0120^T^ lacks any homologs of MCK8115391 ammonium transporter protein. Its function could be performed by a less selective transporter protein MCL4078770 sharing weak but considerable homology (30% sequence identity at an e-value 0.0001) with AmtB protein of *Azospirillum brasilense* belonging to 1.A.11 ammonium channel transporters family (according to Transporter Classification Database^4^, Saier et al., 2021).

### Hydrogen metabolism

The genomes of strains M08DHB^T^ and Es71-Z0120^T^ contain a gene cluster encoding membrane-bound hydrogenase belonging to the Group 1i respiratory H_2_-uptake [NiFe] hydrogenases of *Coriobacteriia-*type, according to the HydDB classifier^5^. This gene cluster encodes large and small hydrogenase subunits, cytochrome *b* subunit and a hydrogenase maturation protease (MCK8113990-2; MCL4078930-2). [NiFe] 1i hydrogenases have been found exclusively in *Actinomycetota* of the order *Coriobacteriales* and likely mediate hydrogenotrophic respiration using cytochrome *b* and quinone with an unresolved electron acceptor (Søndergaard et al., 2016). The genomes of both strains also encode several hydrogenase-related proteins HypABCDEF involved in metallocenter assembly (MCK8114656-7; MCK8115855-9; MCL4078352-4; MCL4079307-8). In addition, only the genome of strain M08DHB^T^ encode a [NiFe] hydrogenase belonging to the Group 3b. Some complexes of this cytoplasmic bidirectional NADP-coupled enzyme are proposed to harbor sulfhydrogenase activity, reducing elemental sulfur with molecular hydrogen (Ma et al., 1993). We identified a gene cluster (MCK8114995-8) encoding alpha and delta hydrogenase subunits and beta and gamma sulfur reductase subunits of a sulfhydrogenase complex. Beta and gamma subunits of the sulfur reductase from strain M08DHB^T^ have 32 and 40% amino acid sequence identity with HydB and HydG of the archaeon *Pyrococcus furiosus*, respectively. MCK8114997 protein contains FAD/NAD-binding pocket, and MCK8114998 one contains 4Fe-4S dicluster domain. Thus, the ability of strain M08DHB^T^ to grow hydrogenotrophically with S^0^ as an electron acceptor may be provided by the action of the sulfhydrogenase.

### Fe(III) reduction

Both isolates possess wide repertoire of multiheme *c*-type cytochromes with predicted membrane bound or cell surface-associated localizations. The genomes of strains M08DHB^T^ and Es71-Z0120^T^ encode 18 and 17 multihemes, respectively. Their genes are scattered along the genomes, although some clusters could be emphasized. One of these clusters in the strain M08DHB^T^ (loci MCK8114672-75) encodes homologs of outer membrane-associated Fe(III)-reducing multihemes MtrA of *Shewanella oneidensis* (White et al., 2016) and OmcH of *Geobacter sulfurreducens* (Aklujkar et al., 2013), as well as a homolog of putative terminal Fe(III) reductase CFE_2239 identified in *Carboxydocella thermautotrophica* (Toshchakov et al., 2018). Another gene cluster of the strain M08DHB^T^ encode weak homologs of MtrA and OmcH together with cytochrome *c* biogenesis proteins (loci MCK8114945-47), the homologs of MtrA, putative terminal Fe(III) reductase OcwA of ‘*Thermincola potens*’ (Costa et al., 2019), and a rubrerythrin (redoxin) protein (loci MCK8115166-68). An additional cluster encodes two putative quinol oxidizing multihemes related to Ga0395992_02_135863_137242 of *Carboxydothermus ferrireducens* (Gavrilov et al., 2021) together with an MtrA homolog (loci MCK8115470-72). Interestingly, only the last gene cluster is reproduced in the genome of strain Es71-Z0120^T^ (loci MCL4078710-12), while other multiheme genes of this Fe(III) reducing strain are combined in two peculiar clusters. The first cluster (MCL4079323-32) encodes homologs of MtrA, OmcH and a quinol oxidizing multiheme of *C. ferrireducens* together with cytochrome *c* biogenesis proteins and a Rex-type redox-sensing suppressor which genes are absent from M08DHB^T^ genome. The second cluster (MCL4079376-90) encodes homologs of the same quinol oxidizing multiheme and the CFE_2239 multiheme, as well as a molybdopterin oxidoreductase and a weak homolog of SmhB cytochrome, involved in soluble ferric citrate reduction in *C. ferrireducens* (Gavrilov et al., 2021). Simultaneous involvement of membrane-bound multiheme cytochrome *c* and molybdopterin enzyme complexes in Fe(III) oxide reduction has been recently proposed for the archaeon *Pyrodictium delaneyi* (Kashyap and Holden, 2021). Probably, peculiar organization of the cluster MCL4079376-90 in the strain Es71-Z0120^T^ is the main feature determining the Fe(III) reducing ability in contrast to its non-Fe(III) reducing counterpart.

### Protocatechuate catabolism

Strain M08DHB^T^ was isolated under anaerobic conditions with 3,4-dihydroxybenzoic acid and sulfate. Further experiments demonstrated its inability to metabolize sulfate as the electron acceptor, indicating protocatechuic acid involvement in energy and carbon metabolism of the organism. Genome analysis revealed the absence of key enzymes (protocatechuate-3,4-dioxygenase and protocatechuate-4,5-dioxygenase) for aerobic catabolism of protocatechuate and catechol in strains M08DHB^T^ and Es71-Z0120^T^. We also did not detect any of the genes encoding 3-hydroxybenzoate-CoA ligase and 4-hydroxybenzoyl-CoA reductase of peripheral pathways for anaerobic degradation of aromatic compounds in both strains. The ABC subunits of benzoyl-CoA reductase (BcrABCD) from *Thauera aromatica* show 54–67% similarity to the two-component acyl-CoA dehydratase activase/2-hydroxyacyl-CoA dehydratase (HAD; MCK8115292-3) of strain M08DHB^T^. HAD belongs to the same radical enzyme family as Bcr-type benzoyl-CoA reductases and is typical for glutamate-fermenting *Bacillota* and *Fusobacteriota* (Buckel et al., 2014). Since M08DHB is not able to grow on glutamate, we suppose that the protein MCK8115292-93 can be involved in the degradation of aromatics. In addition, three genes from strain M08DHB^T^ genome are similar to *bamB*, *bamC* and *bamD* genes of a distinct benzoyl-CoA reductase from *Geobacter metallireducens* (Wischgoll et al., 2005). However, these genes are not co-localized in M08DHB^T^.

### Sulfur catabolism

Besides the sulfhydrogenase complex (see above), the genome of strain M08DHB^T^ encodes three subunits of a complex iron–sulfur molybdoenzyme (CISM, Rothery et al., 2008), namely: a molybdopterin cofactor-containing catalytic subunit (MCK8114746), a four-cluster protein subunit that contains 4 [Fe-S] clusters (MCK8114747), and a membrane anchor protein, containing a diheme *b*-binding PF01292 domain. Amino acid sequence analysis of these three proteins revealed their highest similarity with the subunits of predicted FdnGHI-type formate dehydrogenases (max. 52% sequence identity). These enzymes are distantly related to thiosulfate/polysulfide reductases of PhsA/PsrA group, and further phylogenetic analysis of the proteins from strain M08DHB^T^ is needed to correctly predict the exact function of its CISM family complex. However, characteristic features of the membrane anchor protein MCK8114748 (4 predicted transmembrane helices and the presence of the diheme *b*-binding conserved domain) indicate similarity of the oxidoreductase complex with thiosulfate/polysulfide reductases (Rothery et al., 2008). This fact together with the absence of other determinants of thiosulfate reduction in the genome of M08DHB^T^ allows us to propose the key role of the MCK8114746-8 protein complex in thiosulfate, and probably sulfur, respiration. Genome of strain Es71-Z0120^T^ encodes only a molybdopterin-containing catalytic domain of CISM family oxidoreductases (MCL4079383) presumed to be a part of the formate dehydrogenase. The other genes typical for CISM-family complexes are absent in the organism that is consistent with its inability to reduce sulfur or thiosulfate.

## Discussion

We have isolated in a pure culture two strains of anaerobic bacteria belonging to so far uncultured actinobacterial OPB41 group. The habitats of both isolates are related to deep subsurface. The mud volcanoes of the Taman Peninsula and aquifers of the YMWD are located 500 km apart from each other and were formed during the Alpine geosyncline (Tveritinova et al., 2015). A common feature of these environments is the presence of a gas-water fluid which, however, has different origins. In mud volcanoes of the Taman Peninsula, feeder channels penetrate the Cenozoic sediments and approach the Cretaceous and Jurassic sedimentary rock strata, where the gas-water fluid is formed (Kholodov, 2019). The source of gas (methane) is the buried organic matter transformed at high pressure and temperature. In YMWD, the gas-water fluid flows from the crystalline basement through vertical fractures and cracks, and the major gas component is carbon dioxide with traces of molecular hydrogen of juvenile origin (Lavrushin et al., 2020). Both regions are characterized by oil and gas-bearing manifestations (Tari et al., 2021). The survey of the environmental distribution of OPB41-related phylotypes, and in particular, of the phylotypes related to the proposed ‘*Anaerosomataceae’* lineage within the OPB41 group (Figure 1), shows that these actinobacteria are generally cosmopolitan. However, they are wider represented in oil-bearing environments, subsurface and various sedimentary ecosystems (Supplementary Figures S3, S4). Only 2 of 106 OPB41-related 16S rRNA sequences and MAGs, considered for our analysis, were retrieved from endosymbiotic microbial communities (from a termite gut and a sphagnum moss) indicating preferably free-living lifestyle of these actinobacteria.

OPB41 group belongs to the class *Coriobacteriia* which is currently subdivided into two orders, *Coriobacteriales* and *Eggerthellales*. Majority of *Coriobacteriia* species are the components of normal enteric microbiomes. The only and the type species of the genus *Coriobacterium, C. glomerans*, is an endosymbiont of pyrrhocorid bugs. *Eggerthella lenta* is a member of the normal human intestinal microbiome and has been most commonly associated with infections from a gastrointestinal tract. In contrast, our isolates are free-living bacteria, and the majority of OPB41-related phylotypes are also likely to represent free-living organisms. Our isolates have only several features widespread among *Coriobacteriia*: they are Gram-positive non-motile strictly anaerobic non spore-forming neutrophilic rods (Table 2). The common physiological characteristic of strains M08DHB^T^ and Es71-Z0120^T^ is their ability to grow lithotrophically on molecular hydrogen. This capability indicates that OPB41 bacteria are likely involved in primary organic matter production. Such ecological role is unusual for *Coriobacteriia*, among which fermentative species predominate. Only one member of this class, *Denitrobacterium detoxificans*, has been reported to oxidize H_2_ with trimethylamine oxide or dimethyl sulfoxide as an electron donor in the presence of ruminal fluid (Anderson et al., 2000). Our genomic analysis suggests that during hydrogenotrophic growth, strains M08DHB^T^ and Es71-Z0120^T^ could assimilate CO_2_ *via* reductive glycine pathway. This seventh metabolic route of inorganic carbon fixation in prokaryotes has been poorly investigated, and so far, has been proposed or experimentally confirmed only in a limited number of microorganisms belonging to *Pseudomonadota* (of the former class *Deltaproteobacteria*) and *Bacillota* (Figueroa et al., 2017; Sánchez-Andrea et al., 2020; Song et al., 2020).

**Table 2:** Comparative characteristics of novel isolates and type representatives of the сlass *Coriobacteriia*.

Features	1	2	3	4
Cell morphology	Straight to slightly curved singular rods	Straight to slightly curved singular rods	Pear-shaped to irregularly shaped rods, forming chains	Rod-shaped, occur singly, in pairs, or in short chains
Motility	–	–	–	–
Relation to oxygen	Obligate anaerobe	Obligate anaerobe	Obligate anaerobe	Obligate anaerobe
Temperature optimum, ^0^C	30	47–60	30	37
pH optimum	7.0–7.5	6.5–7.0	6.5	7.7
Metabolism:				
Fermentation of organic compounds	+ grows with protocatechuate and yeast extract without electron acceptors	–	+ saccharolytic	+ saccharolytic, proteinolytic
Electron acceptors	Sulfur, thiosulfate	Fe(III)-citrate, Fe(III) oxide	–	–
Electron donors for respiration	+ formate, H_2_	+ formate, H_2_	–	–
Major cellular fatty acids	C18:0, C16:0, C18:1 n-9	C18:2 n-6, C18:1 n-9, C16:0	ND*	ai-C15:0, C16:0, i-C14:0, C18:1 n-9
Quinones	Not detected	Not detected	ND*	Menaquinone (MK-6), methylated menaquinone (MMK-6)
G + C, mol%	66.58	67.65	61	64.2
Isolation source	Terrestrial mud volcano	Deep subsurface mineral water aquifer	Intestinal tract of red soldier bug (*Pyrrhocoris apterus* L.)	Human faeces

* *ND –* not determined.

1 – strain M08DHB^T^ (this study); 2 – strain Es71-Z0120^T^ (this study); 3 – *Coriobacterium glomerans*, type species of the order *Coriobacteriales* (Haas and König, 1988; Gupta et al., 2013); 4 –*Eggerthella lenta*, type species of the order *Eggerthellales* (Eggerth, 1935; Kageyama et al., 1999; Gupta et al., 2013).

The main physiological differences between strains M08DHB^T^ and Es71-Z0120^T^ include thermophily, the ability to grow on aromatic compounds and the spectrum of utilized electron acceptors. Strain M08DHB^T^ is mesophilic, while strain Es71-Z0120^T^ is a true thermophile with a broad temperature range for growth (25–77°C). Thus, different representatives of OPB41 group can proliferate at moderate as well as at elevated temperatures, which is consistent with molecular data on their environmental distribution (Supplementary Figures S3, S4).

Only strain M08DHB^T^ can grow on 3,4-dihydroxybenzoic acid. This compound, with a trivial name protocatechuate, could be formed during aerobic or anaerobic degradation of lignin-associated phenolic compounds (Philipp et al., 2002; Linger et al., 2014; Upadhyay and Lali, 2021). About 10 to 20% of petroleum is composed of hydrocarbons originating either from biosynthetic processes or from organic matter transformation during diagenesis, which gives grounds to consider plant lignin as the plausible source of petroleum (Libes, 2009). All the currently reported lignin-degrading bacteria belong to aerobic *Actinomycetota*, *Pseudomonadota*, or *Bacillota* phyla (Bugg et al., 2011). Information about the organisms anaerobically degrading lignin-associated or any other aromatic compounds is scarce, and no data on anaerobic degradation of aromatics by *Actinomycetota* has been reported so far. Analysis of strain M08DHB^T^ genome did not reveal any complete aerobic or anaerobic pathways of aromatic compounds degradation, but some crucial determinants of protocatechuate oxidation has been identified. Further investigations are needed to uncover biochemical routes of aromatic compounds transformation in the strain M08DHB^T^ and *Coriobacteriia*, in general.

Peculiar metabolic feature of strain Es71-Z0120^T^ is its obligate dependence on Fe(III) as the electron acceptor for growth, that is a rare case among dissimilatory iron-reducing microorganisms. In contrast, strain M08DHB^T^ does not use Fe(III) as the electron acceptor. However, both isolates possess similar set of 17 genes encoding multiheme *c*-type cytochromes, which are regarded to be the major determinants of extracellular electron transfer to Fe(III) compounds in various prokaryotes (Shi et al., 2016). These genes are differentially clustered with each other and regulatory regions in the genomes of two strains. Imperfect gene clustering, i.e., caused by random mutations, could be the main reason for the inability of strain M08DHB^T^ to reduce Fe(III). Alternatively, the cytochromes of M08DHB^T^ isolate might have high specificity to a particular Fe(III) form, different from ferric oxides or soluble complexes, e.g., to Fe(III) silicate minerals. Genomic background of the inability of Es71-Z0120^T^ strain to reduce sulfur or thiosulfate is clearer. This strain, in contrast to M08DHB^T^, lacks the genes of iron–sulfur and membrane anchor subunits of CISM family enzyme complexes to which thiosulfate/polysulfide reductases belong. The specificity of our isolates to electron acceptors correlates with different representation of sulfur and iron compounds in their natural environments. Soluble iron but not sulfur compounds were detected in mineral waters used for the isolation of the Fe(III)-reducing strain Es71-Z0120^T^, while no iron but 1.2 mM sulfate was detected in the samples taken for the isolation of the non-iron-reducing strain M08DHB^T^, capable of sulfur and thiosulfate respiration. Accordingly, the availability of inorganic electron acceptors seems to be an important selective factor driving the evolution of metabolic capacities within the OPB41 group actinobacteria. This fact and the capability for lithotrophic growth with formate or hydrogen are novel cases for the organisms of *Coriobacteriia* class which has been previously represented mainly by endosymbiotic organotrophs. This work expands the knowledge of the diversity, metabolic functions and ecological role of the phylum *Actinomycetota*. Based on phylogenetic position, phenotypic, physiological and genomic properties of strains M08DHB^T^ and Es71-Z0120^T^, we propose to assign them to the novel taxa of the species–order level.

### Description of *Anaerosoma* gen. nov.

*Anaerosoma* (An.ae.ro.so’ma. Gr. pref. *an*-without; Gr. masc. n. *aer* – air; Gr. neut. n. *soma* - body; N.L. neut. n. *Anaerosoma*, anaerobic body).

Non-motile, slightly curved to rod-shaped bacterium. Mesophilic, obligate anaerobe, catalase- and oxidase-positive. Non spore-forming. Gram-positive. Elemental sulfur or thiosulfate are used as the electron acceptors and reduced to hydrogen sulfide. Molecular hydrogen and formate can be used as electron donors. Unable to ferment carbohydrates and proteins. The major fatty acids are С18:03, C16:0 and С18:1 n-9. Quinones were not detected. Member of the family *Anaerosomataceae*, order *Anaerosomatales*, class *Coriobacteriia*. The type species is *Anaerosoma tenue*.

### Description of *Anaerosoma tenue* sp. nov.

*Anaerosoma tenue* (te’nu.e. L. neut. Adj. *tenue* – small, slender).

Nonmotile, rod-shaped bacterium with pili-like appendages, 0.8–1.4 μm in length and 0.14–0.18 μm in diameter. Strictly anaerobic, but catalase- and oxidase-positive. Non-spore-forming. Gram-positive type of the cell wall. Grows at 14–42°C (optimum 30°C), at pH 6.0–8.5 (optimum 7.0–7.5) and at NaCl concentrations of 0–70 g l^−1^ (optimum 5.0–10 g l^−1^). Yeast extract is necessary for growth. Grows on 3,4-dihydroxybenzoic acid only in the presence of yeast extract, producing acetate, CO_2_, and traces of H_2_. Is able to reduce elemental sulfur or thiosulfate as the electron acceptor with formate or molecular hydrogen as the electron donor. Products of chemolithotrophic growth are carbon dioxide and hydrogen sulfide. No growth is observed on D-glucose, D-fructose, D-mannose, D-ribose, D-sucrose, lactose, cellobiose, succinate, pyruvate, malate, fumarate, lactate, galactose, arabinose, citrate, acetate, yeast extract, tryptone, peptone, methanol, ethanol, trimethylamine, phenol, lactate, butyrate, glycerol, glutamate, benzoate, 2-methoxyphenol, 2-methoxybenzoate, 3,4-dimethoxybenzoate, vanillate, 2-methoxycinnamic acid, 2-hydroxybenzoate, 3-hydroxybenzoate, 2,5-dihydroxybenzoate, 2-hydroxycinnamic acid, 4-aminobenzoate, 2-chlorobenzoate. Ferrihydrite (poorly crystalline Fe(III) oxide), sulfate, nitrate, nitrite, sulfite, AQDS, dimethylsulfoxide, arsenate, selenate, selenite, crotonate are not reduced and do not support growth. The genome of the type strain is characterized by the size of 2.11 Mb and a G + C content of 66.6%. The type strain, M08DHB^T^ (=VKM B-3570^T^ = JCM 39237^T^ = KCTC 25380^T^ = UQM 41472^T^), was isolated from terrestrial mud volcano, Taman Peninsula, Russia.

### Description of *Parvivirga* gen. nov.

*Parvivirga* (Par.vi.vir’ga. L. masc. Adj. *parvus*, tiny, small; L. fem. n. *virga*, rod; N.L. fem. n. *Parvivirga*, a tiny rod).

Non-motile, slightly curved to rod-shaped bacterium. Non spore-forming. Gram-positive. Obligate anaerobe, positive for catalase and oxidase. Neutrophile and thermophile. Chemolithotrophic growth is possible with molecular hydrogen or formate as the electron donors. Capable of dissimilatory Fe(III)-reduction. Unable to ferment carbohydrates and proteins. The major fatty acids are C18:2 n-6, C18:1 n-9, C16:0. Respiratory quinones were not detected. Member of the family *Anaerosomataceae*, order *Anaerosomatales*, class *Coriobacteriia*. The type species is *Parvivirga hydrogeniphila*.

### Description of *Parvivirga hydrogeniphila* sp. nov.

*Parvivirga hydrogeniphila* (hy.dro.ge.ni’phi.la. N.L. neut. n. *hydrogenum*, hydrogen; N.L. masc. Adj. *philus* (from Gr. masc. adj. philos), loving; N.L. fem. Adj. *hydrogeniphila*, hydrogen-liking, referring to its ability to grow lithotrophically on molecular hydrogen).

Non-motile or slightly curved small single rods, 0.5–1.0 μm in length and 0.12–0.18 μm in width. In the presence of ferrihydrite, cells form exocellular, long pili-like appendages, 0.8–0.9 nm in diameter. Non spore-forming. Gram-positive type of the cell wall. Strictly anaerobic, but catalase- and oxidase-positive. Thermophile, grows at 25–70°C (optimum 47–60°C) at pH 6.0–8.5 (optimum 6.8–7.2), at NaCl concentrations of 0–35 g l^−1^ (optimum at 0–0.5 g l^−1^) and at NaHCO_3_ concentrations of 0–10 g l^−1^ (optimum 2.0 g l^−1^). Vitamins, but not yeast extract are necessary for growth. Chemolithotroph, capable of growth using molecular hydrogen or formate as the only electron donor with synthetic ferrihydrite (SF) or Fe(III)-citrate as the electron acceptors. The products of chemolithotrophic growth are carbon dioxide and ferrous iron in the form of siderite or magnetite. Unable to oxidize methanol, ethanol, *n*-propanol, butanol, acetate, lactate, pyruvate, succinate, malate, citrate, microcrystalline- or carboxymethylcellulose, N-acetyl-D-glucosamine in the presence of SF or Fe(III)-citrate as the electron acceptors. Cannot ferment peptone, yeast extract, beef extract, tryptone, D-glucose, D-sucrose, cellobiose. Does not grow on 3,4-dihydroxybenzoic acid, whether in the presence or absence of yeast extract. Sulfate, sulfite, thiosulfate, elemental sulfur, dimethylsulfoxide, nitrate, nitrite, arsenate, selenate, selenite, crotonate, fumarate, Fe(III)-EDTA, AQDS are not reduced and do not support growth. The genome of the type strain is characterized by the size of 1.84 Mb and a G + C content of 67.7%. The type strain, Es71-Z0120^T^ (=VKM B-3556^T^ = JCM 39246^T^), was isolated from subsurface mineral water of Yessentukskoye mineral water deposit (production well 71), Stavropol Krai, Russia.

### Description of *Anaerosomataceae* fam. nov.

*Anaerosomataceae* (An.ae.ro.so.ma.ta.ce’ae. N.L. neut. n. Anaerosoma, a bacterial genus; −*aceae* ending to denote a family; N.L. fem. pl. n. *Anaerosomataceae,* the Anaerosoma family).

Cells are Gram-stain positive, thin rods, non spore-forming. Cells are nonmotile, possess pili-like appendages. Mesophilic and thermophilic. Chemolithotrophic and chemoorganotrophic, asaccharolytic. Respiratory quinones were not detected. The type genus is *Anaerosoma*. The family belongs to the order *Anaerosomatales* of the class *Coriobacteriia*.

### Description of *Anaerosomatales* ord. nov.

*Anaerosomatales* (An.ae.ro.so.ma.ta’les. N.L. neut. n. Anaerosoma, a bacterial genus; −ales ending to denote an order; N.L. fem. pl. n. *Anaerosomatales*, the Anaerosoma order).

Cells are Gram-stain positive, rod-shaped, anaerobic. The type genus is *Anaerosoma*. The order contains the single family *Anaerosomataceae* and belongs to the class *Coriobacteriia*.

## Data availability statement

The datasets presented in this study can be found in online repositories. The names of the repository/repositories and accession number (s) can be found at: https://www.ncbi.nlm.nih.gov/genbank/, ON668121, OP389241, JALNTY010000000, JAMCCO010000000.

## Author contributions

MK, DZ, AM, AK, and VP: experimental work. AS, MK, AM, and SG: genome annotation and analysis. MK, DZ, and SG: writing – original draft preparation. AS, MK, and SG: writing –review and editing. MK, DZ, and AM: visualization. AS and SG: funding acquisition. All authors contributed to the article and approved the submitted version.

## Funding

This research was partially funded by the Russian Science Foundation, grant number 22–14-00011 (MK, AM, AS, isolation and characterization of strain M08DHB), and number 21–14-00333 (DZ, SG, VP, isolation, sequencing and characterization of strain Es71-Z0120), and by the Ministry of Science and Higher Education of the Russian Federation (M08DHB genome sequencing and annotation).

## Conflict of interest

The authors declare that the research was conducted in the absence of any commercial or financial relationships that could be construed as a potential conflict of interest.

## Publisher’s note

All claims expressed in this article are solely those of the authors and do not necessarily represent those of their affiliated organizations, or those of the publisher, the editors and the reviewers. Any product that may be evaluated in this article, or claim that may be made by its manufacturer, is not guaranteed or endorsed by the publisher.

## Footnotes

^1^ https://www.arb-silva.de

^2^ https://gtdb.ecogenomic.org

^3^ http://www.ezbiocloud.net

^4^ https://www.tcdb.org/

^5^ https://services.birc.au.dk/hyddb/
