# Supplementary material for: The first cultivated representatives of the actinobacterial lineage OPB41 isolated from subsurface environments constitute a novel order Anaerosomatales
Source: Front Microbiol. 2022 Nov 10;13:1047580. doi: 10.3389/fmicb.2022.1047580 (PMC9686372; doi:10.3389/fmicb.2022.1047580)
Supplement: Supplementary file 1 [file Data_Sheet_1.docx]

**The First Cultivated Representatives of the Actinobacterial Lineage OPB41 Isolated from Subsurface Environments Constitute a Novel Order *Anaerosomatales***

Maria A. Khomyakova, Daria G. Zavarzina, Alexander Y. Merkel, Alexandra A. Klyukina, Valeria A. Pikhtereva, Sergey N. Gavrilov and Alexander I. Slobodkin

**SUPPLEMENTARY MATERIALS**

**Supplementary Figure S1.**

**16S rRNA gene-based profiling of environmental samples and enrichment cultures from Taman Peninsula and Yessentukskoye mineral water deposit.**


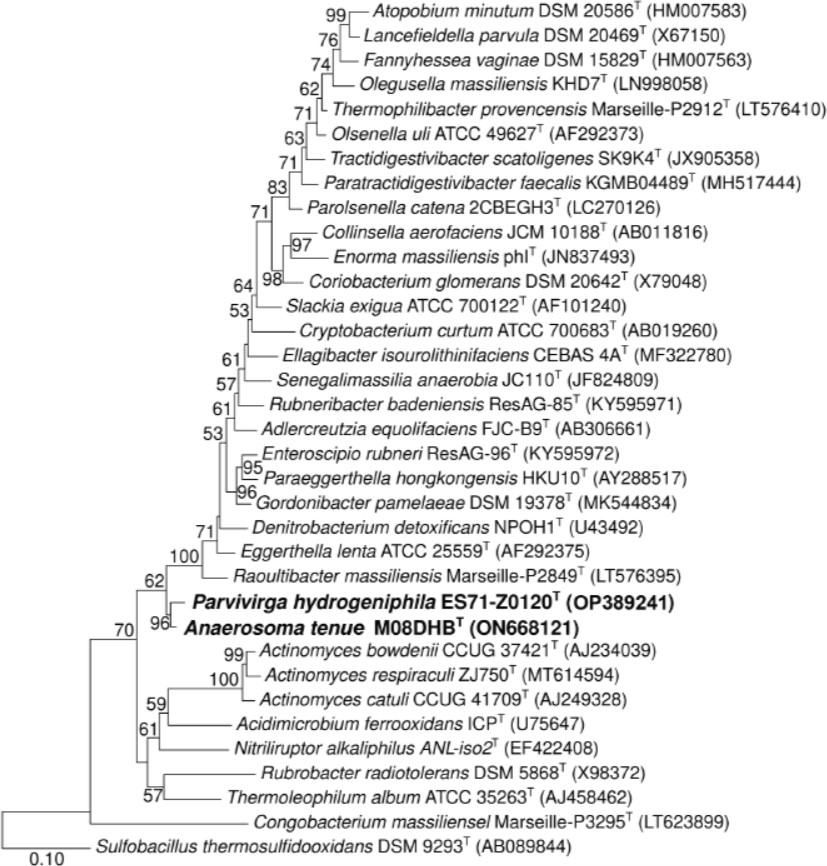


**Supplementary Figure S2.**

**Phylogenetic position of the first cultivated representatives of the actinobacterial lineage OPB41 based on 16S rRNA gene sequence analysis.**

The trees were built using the IQ-TREE 2 program (Minh et al., 2020) with approximate likelihood-ratio test for branches (Anisimova & Gascuel, 2006). Bootstrap consensus tree is shown with values above 50% placed at the nodes. Bar, 0.10 changes per position.

**Supplementary figure S3.**

**Environmental distribution of *Anaerosomataceae*-related sequences by their detection sites.** *(A)* Complete 16S rRNA gene sequences. *(B)* MAGs. Totally, 147 complete 16S rRNA sequences and 15 MAGs were retrieved from the databases of NCBI portal. Only 59 complete 16S rRNA sequences with available data on their detection sites were considered for this analysis.

**Supplementary Figure S4.**

**Environmental distribution of OPB41 group-related MAGs by their detection sites.**

Totally, 47 OPB41-related MAGs were retrieved from NCBI databases and considered for this analysis.

**
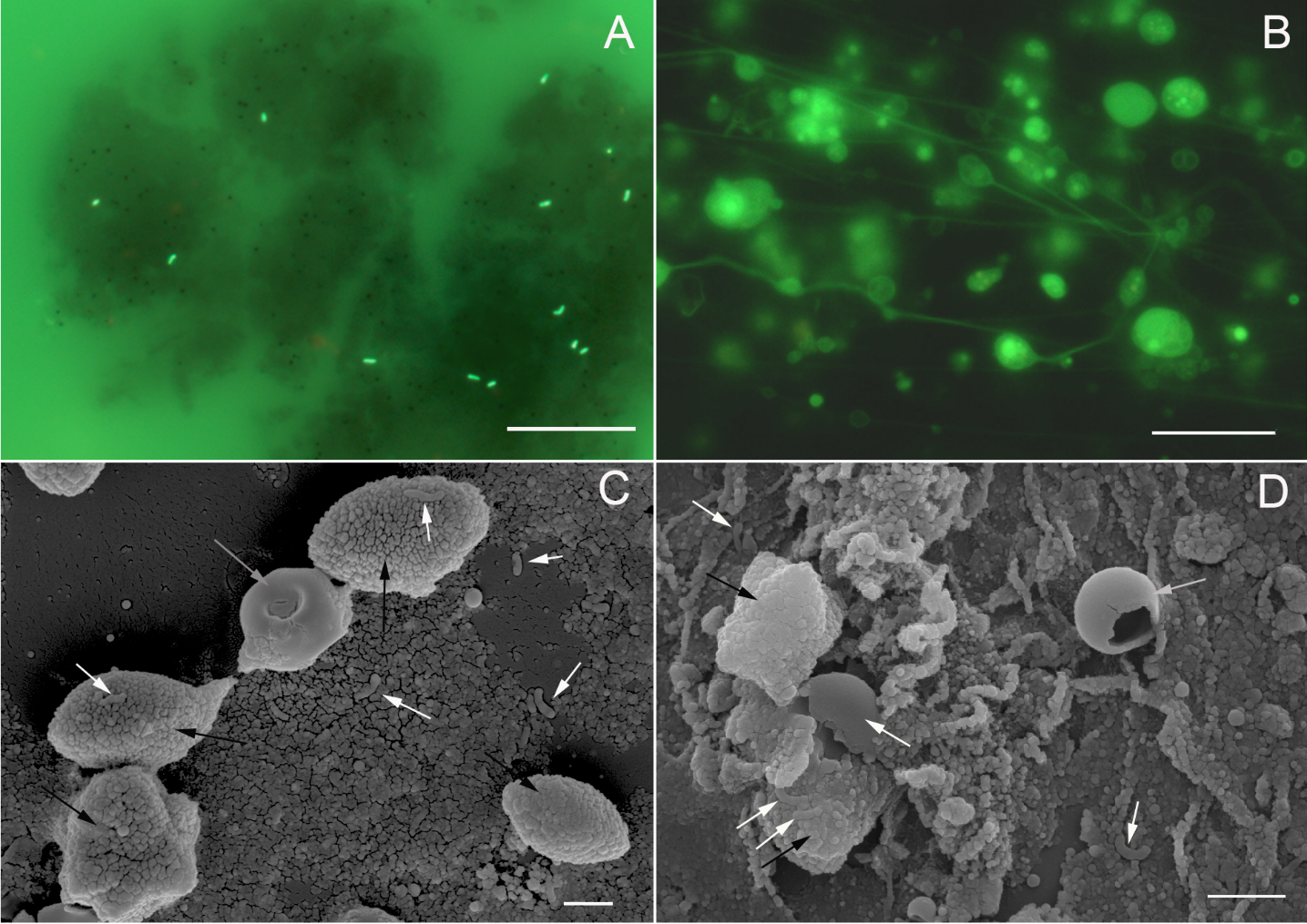
**

**Supplementary Figure S5.**

**Cellular morphology of strain Es71-Z0120^T^.**

*(A)* Fluorescent micrograph of the cells stained with acridine orange colonizing a synthetic ferrihydrite (SF) particle. *(B)* Fluorescent micrograph of acridine-stained dense extracellular matrix with bubble-like structures, formed by the cells in the late stationary growth phase or during a long (4-6 weeks) storage. *(C)* SEM micrograph of siderite crystals produced by the culture of strain Es71-Z0120^T^ during ferrihydrite reduction, *black arrows* indicate the crystals, *white arrows* indicate cells. *(D)* SEM micrographs of the bubble-like structures (*grey arrows*) and siderite crystals (*black arrows*) formed by the cells (*white arrows*) of a ferrihydrite-reducing culture in the late stationary growth phase. Bars: (a, b) =10µm, (c) =1µm, (d) =2µm.


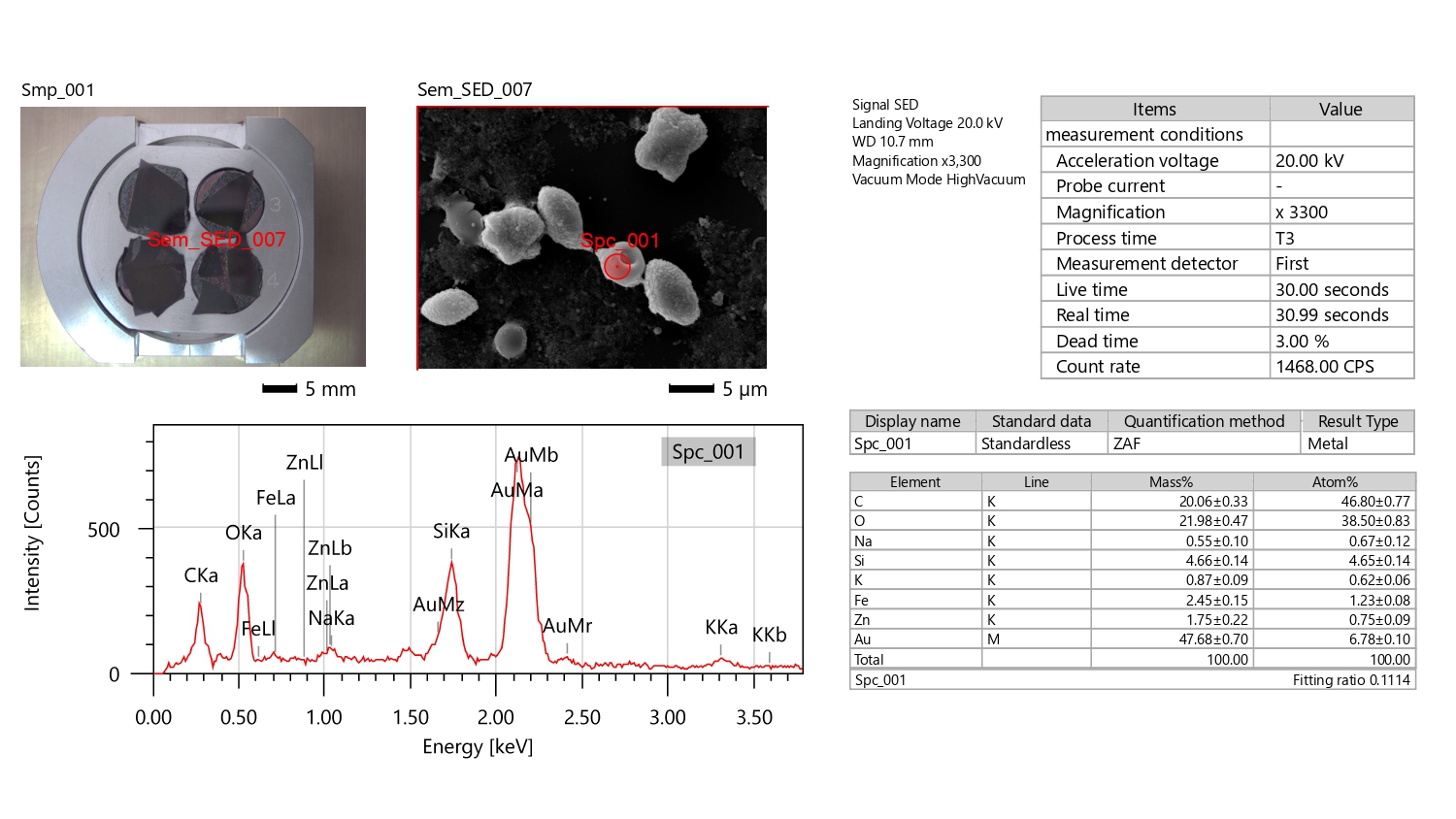

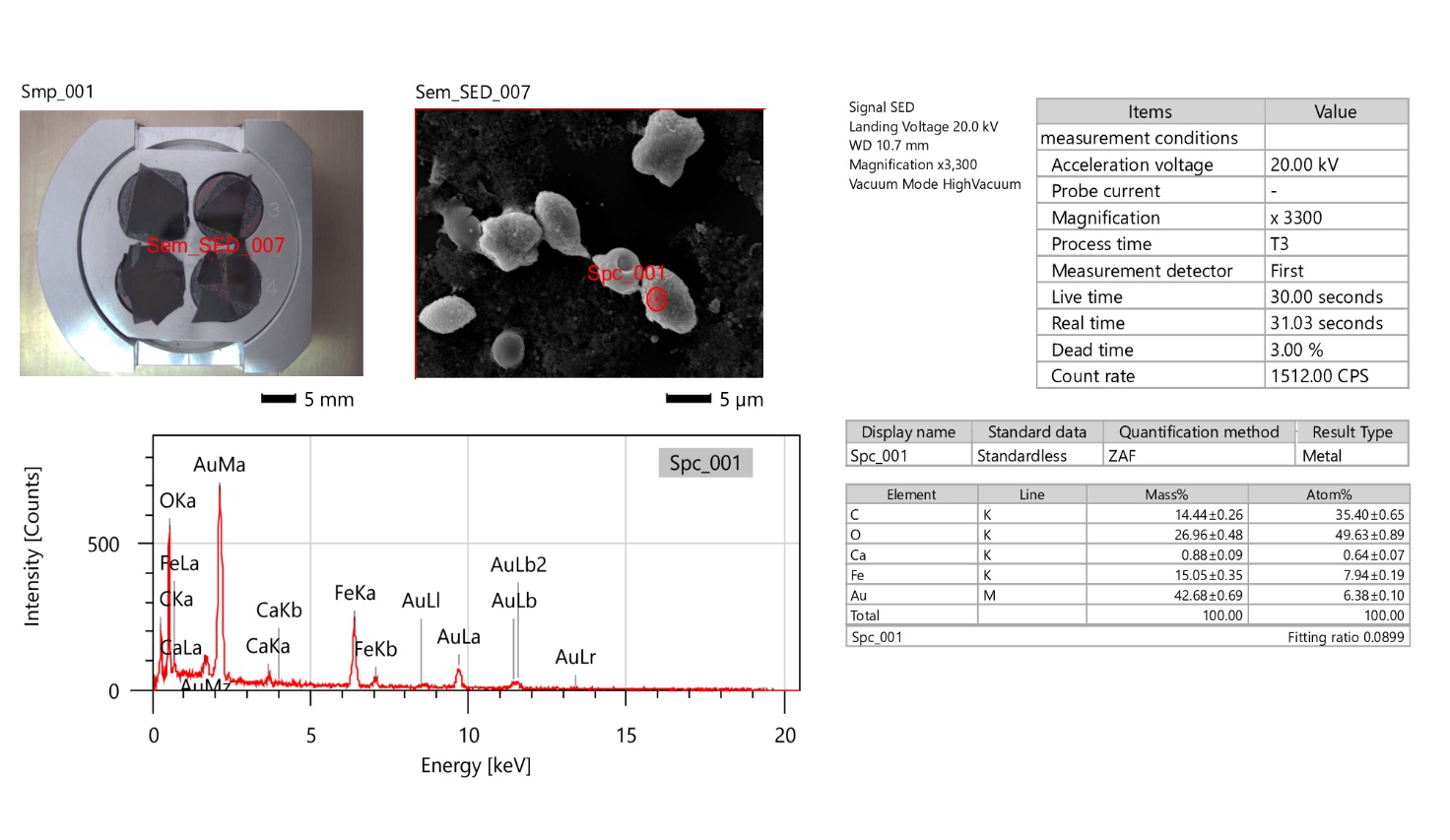


**A**

**B**

**Supplementary figure S6.**

**Elemental composition determined by energy dispersive X-ray spectroscopy of siderite *(A)* and bubble-like structures *(B)* formed by a ferrihydrite-reducing culture of strain Es71-Z0120^T^ in the late stationary growth phase.**

Note the subjects of analysis pointed by the X-ray beam (*red dashed ovals* with “Spc_001” captions) – a rough structure of a siderite crystal on *(A)*, and a smooth structure of a “bubble” near the crystal on *(B)*.

**Supplementary Tables**

Table S1. Cellular fatty acids of two strains of the family *Anaerosomataceae.*

| *C:D* numerical symbol | Strain M08DHB^T^ | Strain Es71-Z0120^T^ |
| --- | --- | --- |
| C10:0 | 0 | 0.07 |
| 9-oxo C10:0 | 1.82 | 0 |
| C12:0 | 0.57 | 0.25 |
| C12:1 n-7 | 0.27 | 0 |
| ai-C13:0 | 0 | 0.19 |
| C14:0 | 2.43 | 1.17 |
| C14:1 n-5 | 0.79 | 0 |
| C15:0 | 0 | 0.91 |
| i-C15:0 | 0 | 2.71 |
| ai-C15:0 | 0 | 3.95 |
| C16:0 | 23.95 | 12.81 |
| C16:0 n-7 | 10.19 | 0.95 |
| C16:1 n-5 | 0 | 0.68 |
| C16:1 n-9 | 1.44 | 0 |
| i-C16:0 | 0 | 0.66 |
| C18:0 | 27.37 | 6.7 |
| C18:1 n-9 | 22.31 | 21.52 |
| C18:1 n-12 | 1.36 | 2.47 |
| C18:2 n-6 | 7.49 | 44.96 |

Table S2. Genome properties of the two strains of the family *Anaerosomataceae.*

| **Attributes** | **Strain M08DHB^T^** | **Strain Es71-Z0120^T^** |
| --- | --- | --- |
| Sequence size (bp) | 2,107,022 | 1,840,996 |
| Number of contigs | 6 | 9 |
| GC content (%) | 66.58 | 67.65 |
| Longest contig size | 1,006,512 | 1,128,135 |
| N50 value | 556,904 | 1,128,135 |
| L50 value | 2 | 1 |
| Total number of genes | 2,100 | 1,808 |
| Total CDSc | 2,047 | 1,754 |
| Pseudogenes | 9 | 26 |
| Number of RNAs (tRNAs, rRNAs, ncRNAs) | 53 (46, 3, 4) | 54 (47, 3, 4) |
| Genbank Accession number | JALNTY010000000 | JAMCCO000000000 |
